# Iron binding before iron limitation: siderophore synthesis arose well before iron became a limiting element

**DOI:** 10.1093/ismeco/ycag025

**Published:** 2026-02-06

**Authors:** Ricardo Soares, Inês B Trindade, Ricardo O Louro

**Affiliations:** Instituto de Tecnologia Química e Biológica da Universidade Nova de Lisboa, Avenida da República (EAN), Oeiras 2780-157, Portugal; Instituto Nacional de Investigação Agrária e Veterinária, Avenida da República, Quinta do Marquês, Oeiras 2780-157, Portugal; Instituto de Tecnologia Química e Biológica da Universidade Nova de Lisboa, Avenida da República (EAN), Oeiras 2780-157, Portugal; Current affiliation: Division of Biology and Biological Engineering, California Institute of Technology, Pasadena, CA 91125, United States; Instituto de Tecnologia Química e Biológica da Universidade Nova de Lisboa, Avenida da República (EAN), Oeiras 2780-157, Portugal

**Keywords:** siderophores, iron acquisition, the great oxidation event (GOE), molecular evolution

## Abstract

It is widely accepted that the use of siderophores, small molecules that bind and solubilize iron, emerged as a response to the dramatic reduction in bioavailability of this metal in aquatic environments caused by precipitation of iron oxides associated with the Great Oxidation Event (GOE). Here, we report a molecular clock analysis of the time of emergence of siderophore biosynthesis and utilization genes that challenges this view and argues for an emergence of these secondary metabolites that largely predates GOE. The emergence date of Non-ribosomal Peptide Synthase Independent Siderophore synthetases is found to predate by more than 1 Gy the emergence date of ferric siderophore reductases and esterases, which in turn also predate the GOE by approximately 1Gy. This temporal gap is surprising given that these enzymes are essential for microorganisms to obtain iron from siderophores. This timing of events raises questions on the original ecological drivers for the emergence of siderophores. We offer an alternative hypothesis for the origin of siderophores which is their use in ferric mineral dissolution to avoid incrustation of neutrophilic iron oxidizers by metabolically generated ferric iron minerals. The observations and hypothesis reported here highlight the importance of environmental microbe-mineral interactions, beyond nutrient acquisition, as critical selective forces in early Earth, and call for a reassessment of the timing and drivers of siderophore evolution.

## Introduction

Secondary metabolites like siderophores perform specialized roles in microbial competition, defense, communication, and adaptation to environmental stresses. Nowadays, siderophores are used by microbes to scavenge iron, an essential, yet often limiting nutrient. Although iron is the fourth most abundant element in the Earth’s crust, its bioavailability is low, being largely present as insoluble mineral forms. This is a challenge for microorganisms, which is especially acute in environments like the open ocean. Siderophore-mediated iron acquisition is considered to have arisen primarily as a microbial strategy to cope with iron scarcity in aquatic environments following the Great Oxidation Event (GOE) [1], but its evolutionary timeline remains poorly resolved. Understanding when and why these secondary metabolites evolved is key to identifying the environmental pressures that shaped early microbial fitness.

## Results and discussion

To address this gap we analyzed the evolutionary history of four enzyme families with clear roles in siderophore biosynthesis and iron release from ferric siderophores: Non-ribosomal Peptide Synthase Independent Siderophore synthetases (NIS), which specifically produce siderophores and operate individually (unlike NRPS enzymes, which synthesize a broad range of secondary metabolites) [2]; Ferric-Siderophore Reductases (FSR) and Siderophore-Interacting Proteins (SIP) which extract the iron from the ferric-siderophore via reduction [3, 4]; and esterases such as ferric enterobactin esterase (FES) [5] that hydrolyze the ferric ligand to release the iron. We excluded siderophore receptors from this analysis due to their broad substrate recognition, which can include unrelated small molecules, making them unreliable markers of siderophore metabolism. By focusing on enzymes with unambiguous roles in siderophore biosynthesis and iron release, we aimed to reconstruct a clear evolutionary trajectory for siderophore function.

**Figure 1:**
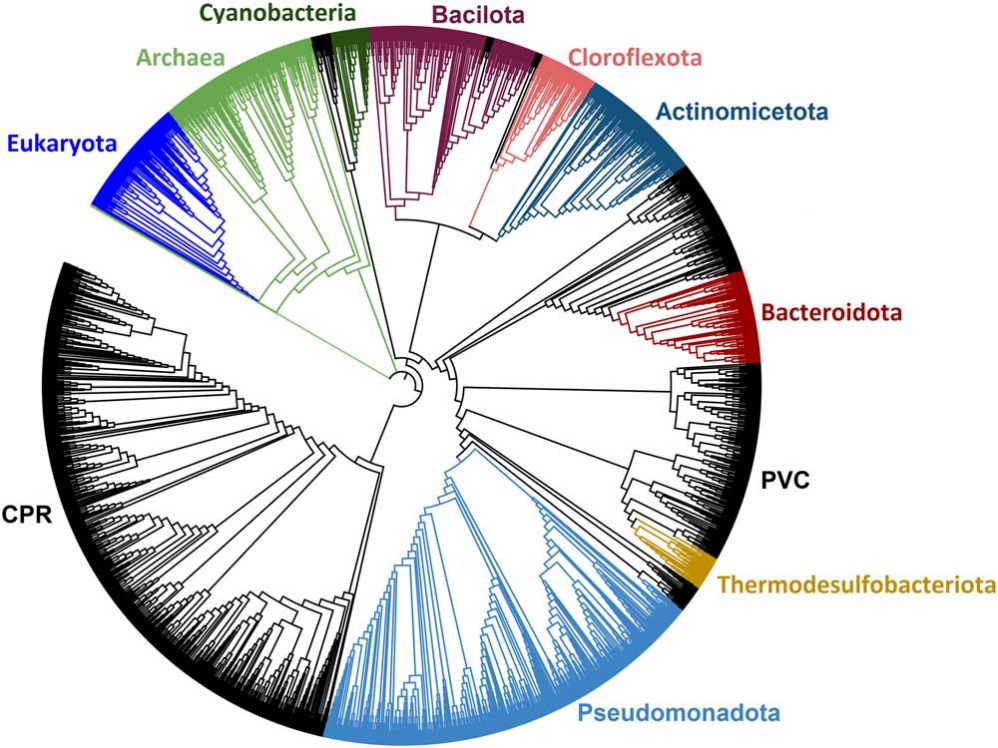
Taxonomic distribution within the tree of life. The distribution within the tree of life [6] of the different enzyme families involved in siderophore synthesis and iron release (NIS, FSR, SIP, FES) highlighted in color. Real branch lengths were not used to facilitate visualization of the whole tree. To avoid cluttering the figure, among the taxonomic groups that do not contain any of these enzyme families (black) only the CPR and PVC are labeled.

**Figure 2:**
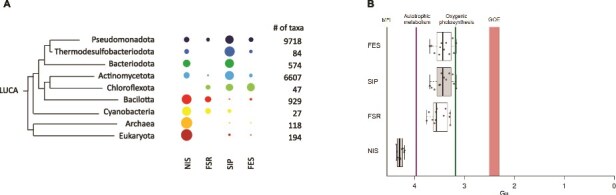
Phylogenetic distribution and chronogram of gene emergence dates. (A) Simplified phylogenetic distribution of the four classes of enzymes (NIS, FSR, SIP, and FES). Only phylogenetic groups where more than 10 taxa contain representatives of at least one of these enzymes are reported. Bubble sizes were normalized for the prevalence of each enzyme in the total number of taxa in each phylogenetic group that contains any of these enzymes, which is indicated on the right-hand side. (B) Chronogram for the appearance of NIS, FSR, SIP, and FES. Interval estimates for the origin of each of the families analyzed. Whiskers plot based on the dated trees of life reported in [7]. Current best estimates for the timing of the moon-forming impact, earliest evidence of autotrophic metabolism and oxygenic photosynthesis and the GOE were obtained from [17–20].

Figure 1 shows that these enzymes are broadly distributed across the Tree of Life, consistent with the universal biological importance of iron. Notable exceptions include the Planctomycetota, Verrucomicrobiota, and Chlamydiota (PVC) and the Candidate Phyla Radiation (CPR) superphyla. These may reflect loss of these systems throughout evolution given that, for example, microorganisms from the CPR have reduced genomes that miss key metabolic genes, in line with the proposal that they are obligate symbionts [6]. Zooming in on the relative taxonomic distribution of the enzyme classes involved in siderophore utilization reveals that NIS and FSR are more ubiquitous across the Tree of Life, being better represented in deep-rooted Bacteria phyla such as Cyanobacteria and Bacilotta (Fig. 2A). NIS are also more abundantly found within Archaea and Eukaryota in comparison with the other SIPs analyzed. In contrast, SIP and FES are more prevalent in more recently diverged phyla, such as Thermodesulfobacteriota and Pseudomonadota, respectively, while they have very little representation on deep rooted phyla such as Cyanobacteria and Bacilliota.

To estimate the timing of origin of each of the corresponding genes, we used calibrated phylogenetic trees reported in the literature [supplementary materials (Table S1)] [7]. Those trees were time calibrated using diverse assumptions including root ages, subsampling of fossil calibrations, alternative topologies, evolutionary models, and scenarios for the dating. Figure 2B plots the gene emergence dates for NIS, FSR, SIP, and FES and shows that the origin of NIS clearly predates the origin of the other three enzyme classes and is likely to have occurred earlier than four billion years ago, in the period known as the Late Heavy Bombardment. The origin of FSR is predicted to have occurred nearly one billion years later, even though these two enzyme classes share the same InterPro signature and are discriminated only by the presence of a specialized binding motif for a 2Fe-2S cluster in the FSRs [4]. The origin of SIPs and FES trailed that of FSRs by about 100 million years and were contemporary within the distribution dispersion of the tree calibrations. This shows that the evolutionary pressure to synthesize siderophores clearly predates the need to scavenge iron from the environment by reduction or hydrolysis of ferric siderophores. Notably, this overall pattern also emerges when using the coarser calibration available from the Time Tree server as an independent control of the relative timing of gene emergence dates. Furthermore, the emergence date of the genes coding for all of these enzymes clearly predates the GOE in all calibration scenarios (Fig. 1 and Fig. S1). Indeed, the origin of these three classes of enzymes predates the GOE by approximately 1 billion years and falls in the period between the origin of anoxygenic photosynthesis and oxygenic photosynthesis. This timing is consistent with recent estimates of the origin of aerobic metabolism to approximately one billion years before the GOE, based on strategies to calibrate the bacterial tree that are different from those used here to calibrate the whole tree of life [8].

These findings challenge the prevailing view for the timing and drivers for the origin of siderophores, which assigns their appearance as a response to a need to scavenge iron in a world where this essential nutrient became scarce due to the GOE [1]. At the time of the appearance of NIS (~4.4 Ga), the global oceans contained abundant ferrous iron and scavenging was unnecessary [9]. This observation supports the following alternative scenario: The competitive advantage provided by siderophores was not the capacity to scavenge iron but the capacity to avoid microbial incrustation by metabolically generated ferric iron minerals. Incrustation by ferric minerals is observed in extant anaerobic iron oxidizers, in particular among photoferrotrophs [10, 11]. Diverse strategies are used to cope with incrustation, such as changes of cell surface potential and hydrophobicity [12], production of organic fibers [13] lowering of surface pH [14], and the use of siderophores that accelerate ferric mineral dissolution via ligand controlled and light induced mechanisms [15].

## Conclusions

The observations presented in this work challenge the current narrative for the timing and evolutionary drivers for the origin of siderophores. It supports a scenario where these metabolites emerged in the presence of abundant ferrous iron in the oceans and in response to the need to avoid incrustation by metabolically generated iron minerals, and not to secure an essential nutrient. Photoferrotrophic iron oxidation provides a metabolic strategy where avoidance of incrustation is competitively advantageous and matches the conditions of the early Earth where oxidation of the oceans proceeded from shallow to deep waters [16]. This observation provides a revised framework for understanding the evolution of microbial iron metabolism.

## Supplementary Material

Supplementary_materials_isme_commun_Review_Comments_clean_ycag025

## Data Availability

All data generated or analysed during this study are included in this published article and its supplementary information files.
