# Supplementary material for: Iron binding before iron limitation: siderophore synthesis arose well before iron became a limiting element
Source: ISME Commun. 2026 Feb 6;6(1):ycag025. doi: 10.1093/ismeco/ycag025 (PMC12919436; doi:10.1093/ismeco/ycag025)
Supplement: Supplementary_materials_isme_commun_Review_Comments_clean_ycag025 [file supplementary_materials_isme_commun_review_comments_clean_ycag025.doc]

**SUPPLEMENTARY MATERIALS**

**Materials and methods**

Sequences for the siderophore interacting proteins and corresponding taxa identifiers (TaxIDs) (see supporting files) were obtained from the InterPro database for all the sequences that were ascribed to each family using all available domain architectures for each family (1). InterPro uses predictive models to define protein families based on common features such as domains, motif fingerprints, sequence profiles, profile Hidden Markov Models (HMMs). These signatures, contributed by several member databases, are unified into nonredundant InterPro entries(2).For NIS and FSR, the InterPro sequence signature of IPR022770 was used. For SIP and FES the InterPro sequence signatures of IPR039374 and IPR021764 were used, respectively (access date: NIS, FSR and SIP - 22/01/2022; FES - 6/02/2022). Since NIS and FSR were mixed within the same signature identifier, as these two families are homologous, iron-sulfur cluster binding motifs were used to separate the FSR family from the remaining SS family. The cluster binding motifs searched were between CCx_2_Cx_2_C to CCx_25_Cx_2_C and CCx_1_Cx_2_C to CCx_25_Cx_1_C, where ‘x’ represents any residue using Microsoft Excel pattern finder with wildcard characters. TaxIDs associated with the sequences belonging to each InterPro signature were obtained from the InterPro “taxonomy-matching” file. Each sequence in InterPro is assigned to a single NCBI TaxID. These TaxIDs were mapped to higher-level taxonomy ranks using the NCBI Common Tree tool within NCBI webserver.(3). This tool retrieves taxonomic information from the NCBI taxonomy and groups the different taxIDs based on their taxonomic distribution. Occurrences at the level of phyla of the different siderophore-interacting proteins were mapped and counted through this tool and topologically organized according to the phylogenetic tree of life (4). Only taxa that contain at least 10 entries for a given family of siderophore-interacting proteins were considered. This cut-off allowed avoiding incongruences resulting from spurious events of horizontal gene transfer or misclassification of hosting organisms. A chronogram was constructed by combining the phylogenetic distribution of the different siderophore interaction proteins obtained previously and the data from the molecular dating of the Tree of Life to determine gene emergence dates using the calibrated trees provided by (5). Within each dated tree of life reconstruction, the most ancient node for each phylogenetic distribution of siderophore interacting proteins was accessed for the gene emergence dates and is presented in supplementary table 1. Boxplots were generated by the BoxPlotR platform (6), and the age values were provided from the different topologies, evolutionary models and scenarios used to date the Tree of Life (5). Alternatively, the emergence of the different families of siderophore-interacting proteins was dated from the different taxonomic distributions using TimeTree 5 (7).

**Table S1: Gene birth date values in millions of years obtained using the calibrated trees provided by (5) using diverse statistical models and evolutionary scenarios.**

|  | **Autocorrelated lognormal (-ln)** | | | | | | **Uncorrelated gamma multiplier (-ugam)** | | | | | |
| --- | --- | --- | --- | --- | --- | --- | --- | --- | --- | --- | --- | --- |
| **Scenarios** | 1 | 2 | 3 | 4 | 5 | 6 | 7 | 8 | 9 | 10 | 11 | 12 |
| **SS** | 4209.03 | 4307.2 | 4365.83 | 4233.04 | 4299.28 | 4257.51 | 4355.24 | 4340.51 | 4304.23 | 4359.27 | 4284.78 | 4201.42 |
| **FhuF** | 3645.55 | 3761.96 | 3575.37 | 3420.46 | 3557.1 | 3717.97 | 3605.53 | 3383.99 | 3289.56 | 3580.32 | 3304.49 | 3294.64 |
| **SIP** | 3606.26 | 3698.23 | 3491 | 3370.84 | 3510.38 | 3664.23 | 3466.96 | 3307.01 | 3214.74 | 3414.43 | 3240.17 | 3167.79 |
| **FES** | 3606.26 | 3698.23 | 3491 | 3370.84 | 3510.38 | 3664.23 | 3466.96 | 3307.01 | 3214.74 | 3414.43 | 3240.17 | 3167.79 |

Figure S1

**Fig S1.** Chronogram for NIS, FSR, SIP and FES- Interval estimates for the emergence date of each of the families analyzed using TimeTree 5 (Kumar et al., 2022). The horizontal line represents the median value of the estimates with the emergence of NIS clearly predating that of the enzymes capable of extracting iron from siderophores. The vertical lines represent 95% confidence intervals of the estimates. Current best estimates for the timing of the Moon-forming impact (MFI), origin of anoxygenic and oxygenic photosynthesis and the GOE were obtained from (8–11).

**References**

1.	Hunter S, Apweiler R, Attwood TK, Bairoch A, Bateman A, Binns D, Bork P, Das U, Daugherty L, Duquenne L, Finn RD, Gough J, Haft D, Hulo N, Kahn D, Kelly E, Laugraud A, Letunic I, Lonsdale D, Lopez R, Madera M, Maslen J, McAnulla C, McDowall J, Mistry J, Mitchell A, Mulder N, Natale D, Orengo C, Quinn AF, Selengut JD, Sigrist CJA, Thimma M, Thomas PD, Valentin F, Wilson D, Wu CH, Yeats C. 2009. InterPro: the integrative protein signature database. Nucleic Acids Res 37:D211–D215.

2.	Blum M, Andreeva A, Florentino LC, Chuguransky SR, Grego T, Hobbs E, Pinto BL, Orr A, Paysan-Lafosse T, Ponamareva I, Salazar GA, Bordin N, Bork P, Bridge A, Colwell L, Gough J, Haft DH, Letunic I, Llinares-López F, Marchler-Bauer A, Meng-Papaxanthos L, Mi H, Natale DA, Orengo CA, Pandurangan AP, Piovesan D, Rivoire C, Sigrist CJA, Thanki N, Thibaud-Nissen F, Thomas PD, Tosatto SCE, Wu CH, Bateman A. 2025. InterPro: the protein sequence classification resource in 2025. Nucleic Acids Res 53:D444–D456.

3.	Federhen S. 2012. The NCBI Taxonomy database. Nucleic Acids Res 40:D136–D143.

4.	Hug LA, Baker BJ, Anantharaman K, Brown CT, Probst AJ, Castelle CJ, Butterfield CN, Hernsdorf AW, Amano Y, Ise K, Suzuki Y, Dudek N, Relman DA, Finstad KM, Amundson R, Thomas BC, Banfield JF. 2016. A new view of the tree of life. Nat Microbiol 1:16048.

5.	Chen S-C, Sun G-X, Yan Y, Konstantinidis KT, Zhang S-Y, Deng Y, Li X-M, Cui H-L, Musat F, Popp D, Rosen BP, Zhu Y-G. 2020. The Great Oxidation Event expanded the genetic repertoire of arsenic metabolism and cycling. Proceedings of the National Academy of Sciences 117:10414–10421.

6.	Spitzer M, Wildenhain J, Rappsilber J, Tyers M. 2014. BoxPlotR: a web tool for generation of box plots. Nat Methods 11:121–122.

7.	Kumar S, Suleski M, Craig JM, Kasprowicz AE, Sanderford M, Li M, Stecher G, Hedges SB. 2022. TimeTree 5: An Expanded Resource for Species Divergence Times. Mol Biol Evol 39:msac174.

8.	Cardona T. 2019. Thinking twice about the evolution of photosynthesis. Open Biol 9:180246.

9.	Jacobson SA, Morbidelli A, Raymond SN, O’Brien DP, Walsh KJ, Rubie DC. 2014. Highly siderophile elements in Earth’s mantle as a clock for the Moon-forming impact. Nature 508:84–87.

10.	Lyons TW, Reinhard CT, Planavsky NJ. 2014. The rise of oxygen in Earth’s early ocean and atmosphere. Nature 506:307–315.

11.	Tashiro T, Ishida A, Hori M, Igisu M, Koike M, Méjean P, Takahata N, Sano Y, Komiya T. 2017. Early trace of life from 3.95 Ga sedimentary rocks in Labrador, Canada. Nature 549:516–518.
